# Rational Truncation of Aptamer for Ultrasensitive Aptasensing of Chloramphenicol: Studies Using Bio-Layer Interferometry

**DOI:** 10.3390/bios13060660

**Published:** 2023-06-16

**Authors:** Richa Sharma, Monali Mukherjee, Praveena Bhatt, K. S. M. S. Raghavarao

**Affiliations:** 1 Department of Food Engineering, CSIR-Central Food Technological Research Institute (CFTRI), Mysore 570020, India; cftri.ftbe.richa@gmail.com; 2 Academy of Scientific and Innovative Research (AcSIR), Ghaziabad 201002, India; monali0812@gmail.com (M.M.); praveena@cftri.res.in (P.B.); 3 Department of Microbiology and Fermentation Technology, CSIR-Central Food Technological Research Institute (CFTRI), Mysore 570020, India; 4 Department of Chemical Engineering, Indian Institute of Technology (IIT)-Tirupati, Tirupati 517619, India

**Keywords:** antibiotics, affinity, biosensing, interferometry, nanoparticles

## Abstract

Aptamers are an excellent choice for the selective detection of small molecules. However, the previously reported aptamer for chloramphenicol suffers from low affinity, probably as a result of steric hindrance due to its bulky nature (80 nucleotides) leading to lower sensitivity in analytical assays. The present work was aimed at improving this binding affinity by truncating the aptamer without compromising its stability and three-dimensional folding. Shorter aptamer sequences were designed by systematically removing bases from each or both ends of the original aptamer. Thermodynamic factors were evaluated computationally to provide insight into the stability and folding patterns of the modified aptamers. Binding affinities were evaluated using bio-layer interferometry. Among the eleven sequences generated, one aptamer was selected based on its low dissociation constant, length, and regression of model fitting with association and dissociation curves. The dissociation constant could be lowered by 86.93% by truncating 30 bases from the 3′ end of the previously reported aptamer. The selected aptamer was used for the detection of chloramphenicol in honey samples, based on a visible color change upon the aggregation of gold nanospheres caused by aptamer desorption. The detection limit could be reduced 32.87 times (1.673 pg mL^−1^) using the modified length aptamer, indicating its improved affinity as well as its suitability in real-sample analysis for the ultrasensitive detection of chloramphenicol.

## 1. Introduction

Chloramphenicol (CAP, Protein Database ID: 1NJI, IUPAC: 2,2-dichloro-n-[1,3-dihydroxy-1-(4-nitrophenyl)propan-2-yl]acetamide) is a bacteriostatic antimicrobial that finds wide application in treatment of acutely infected animals. However, intake of the antibiotic by humans has potential ramifications on health, leading to blood dyscrasias, bone marrow suppression, blue-baby syndrome, and resistance [1]. Thus, the extra-label use of CAP in food-producing animals (such as cattle, poultry, fish, and honeybees) is strictly forbidden under the Animal Medicinal Drug Use Clarification Act [2]. Despite such regulations, CAP has been found in domestic and exported food products, in enzymes added to fodder, and in the environment (animal excreta), creating considerable health and economic losses [3]. Conventional regulatory methods for the monitoring of CAP rely on HPLC-DAD, recommended by the Food and Agricultural Organisation [4]; HPLC-MS-MS, as followed by the Food Safety and Standards Authorisation of India [5]; GC-MS, a technique used by the European Food Safety Association, EU [6]; and LC-MS-MS, commonly practiced by the Food and Drug Administration, USA [7]. These techniques are very accurate; however, they require solvents for the dissolution and flow of analyte through reverse-phase columns, instrumentation and facility set-ups in regulatory labs, and trained personnel to run the operation. They usually possess limited sensitivity; for example, the HPLC-DAD method has a limit of detection of 21.4 ng mL^−1^ in milk samples. Since regulations have a zero-tolerance policy for CAP, detection methods should be able to identify even minute traces.

Aptamers have been proven to be competent bio-recognition and targeting molecules [8]. They provide specificity, tunable synthesis, and modifiability. Several aptasensing platforms have been developed for CAP [9,10,11,12,13,14,15,16,17,18,19,20,21]. However, many of them involve multiple steps, several sets of biomolecules and nanoparticles, and modest sensitivity. Hence, we attempted to optimize a simple visual assay for the screening of CAP [22], in which it competes with nanosphere surfaces to bind to the aptamer. Gold nanospheres were used as the signal transduction element as a result of their strong and stable absorption in the visible spectrum [23,24]. Although the assay responded to low concentrations of CAP in buffer, the biosensing method was not robust, and results were inconclusive. This was because the aptamers failed to show substantial desorption from nanospheres on analyte binding. We hypothesize that this is due to the bulky structure of the aptamer, which prevents efficient target binding, adequate conformational change, and, therefore, insufficient nanoparticle aggregation. This phenomenon was evident from the poor resolution and high blank values in the linear response. Hence, it was important to reduce the aptamer length to ensure definitive response from nanoparticles of aptamer desorption in the presence of analyte.

The present study was, therefore, aimed at improving the sensitivity of the colorimetric aptasensing of CAP by increasing the binding affinity of the aptamer. This is difficult to achieve using de novo aptamer selection [25], although some success was achieved by Duan et al., 2016 [26]. We attempted the rational truncation of the reported aptamer. The designed truncated aptamers were computationally examined for stability and thermodynamic feasibility and subjected to binding affinity studies using biolayer interferometry (BLI). The oligonucleotide with the lowest dissociation constant (K_d_) exhibited a considerable reduction in detection limit in real samples compared to the original aptamer. This was a first attempt to truncate the CAP aptamer to improve bio-affinity and biosensing. This contributes significantly to the development of a simple “yes/no” test for CAP in food samples. In addition, the use of biolayer interferometry for studying nucleic acid-small molecule specificity studies is being reported for the first time.

## 2. Materials and Methods

### 2.1. Materials

Tetrachloroauric acid trihydrate, trisodium citrate, chloramphenicol (CAP), and the original and truncated aptamers (5′ biotin labelled) were procured from Sigma–Aldrich (St. Louis, MI, USA). Dehydrated aptamers were subjected to a brief spin, suspended in water, and stored at −20 °C. The surface-modified probes used for the BLI assay were provided by the manufacturer of the BLI instrument, Octet RED96 (ForteBio, Pall Life Sciences Corp., Menlo Park, CA, USA). Super Streptavidin (SSA) modification is especially suited for small molecular studies. Transmission Electron Micrograph images were obtained using Titan Themis 300 kV, FEI (Thermo Scientific Labs Pvt Ltd., Mumbai, India), housed at the Micro Nano Characterization Facility, the Indian Institute of Science, Bangalore. An HPLC analysis was performed using a Waters HPLC (Quaternary System) with an ELSD detector. The binding buffer for CAP (100 mM NaCl, 20 mM Tris–HCl, 2 mM MgCl_2_, 5 mM KCl, 1 mM CaCl_2_, 0.02 Tween 20, pH 7.6) was used to determine the curves for baseline, association, and neutralization in the BLI protocol, whereas 2 M MgCl_2_ was used for dissociation. All salts used were extra pure and purchased from Ranbaxy Fine Chemicals Limited, Gurgaon, India. MilliQ Millipore plus water was used as a solvent. The software tools used were Mfold (http://unafold.rna.albany.edu/ accessed on 9 August 2016) and the Octet data acquisition software (ForteBio, Pall Life Sciences Corp., Menlo Park, CA, USA).

### 2.2. Methods

#### 2.2.1. Truncation of the Original Aptamer

The truncation design followed the removal of nucleotide stretches (5, 20, 30, and 40 bases) from one or both ends of the original aptamer (80 bases) [27] without interfering with the consensus binding region (Table 1).

#### 2.2.2. Structure, Energetics, and Stability of Truncated Oligonucleotides

The most popular tool for predicting the folding of single stranded oligonucleotides in a solution is Mfold [28]. For the present work, we used sequences, experimental buffer details, and temperature as constraints to obtain secondary structures and circular plots of nucleotide hybridization.

For determining energetics, the curtailed nucleotide sequences and binding conditions ([Na^+^] = 0.1 M and [Mg^2+^] = 0.002 M) were entered as queries in Mfold software. The selected output parameters were melting temperature (Tm), total Gibbs free energy change in folding (ΔG) at 37 °C, enthalpy change (ΔH), and entropy change (ΔS). The program was tuned to give standard errors of approximately ±5%, ±11%, and ±4 °C for ΔG, ΔS, and Tm, respectively [29,30].

#### 2.2.3. Biolayer Interferometry Studies on Binding Affinity

The biotinylated oligonucleotides were suspended in the appropriate buffer and added to a 96-well black microplate (Figure S1 in Supplementary Materials). They were then subjected to interferometric analysis. The BLI analysis follows a series of steps involving the loading of aptamer onto probes, its attachment to analyte, and its subsequent dissociation (refer to Supplementary Information).

#### 2.2.4. Aptasensing of CAP

The aptamer with the highest binding affinity in the BLI studies was selected for a nanosphere aggregation assay of CAP. The original aptamer was used for comparison. Gold nanospheres were synthesized in a lab according to the reported procedure [31] with some modifications. Initial synthesis trials were aimed at identifying the nanosphere size and shape that was advantageous for aggregation-dependent sensing. The final synthesis was performed using the addition of an incubated mixture of 5 mL aqueous tetrachloroauric acid (1 mg mL^−1^) and 1 mL aqueous sodium citrate (10 mg mL^−1^) to 44 mL of boiling water under reflux with constant stirring. Stirring and heating were continued for 25 min to generate the desired shape and size. The dark red resultant colloid was cooled, passed through 1μm filter, and stored at 4 °C.

The assay parameters were optimized following procedures in our previous report [22]. The best designed aptamer was used in these studies and compared with Sequence 1 under the same conditions. The reagents used for aptasensing were an assay buffer (16.67% *v*/*v*), gold nanospheres (1.133 × 10^−9^ M), NaCl (44 mM), and modified aptamer (0.044 μM). The incubation time for binding was 25 min.

The protocol of the aptasensing assay was as follows: Gold nanospheres were incubated with a solution of aptamers in the binding buffer followed by the addition of NaCl. Different concentrations of CAP in the buffer were then added to this, and the solution was mixed thoroughly. The absorption spectra for all the data were recorded and then analyzed.

The incubated nanospheres were protected from salt-induced aggregation via aptamers (red suspension). In the presence of CAP, aptamers selectively bound to their target, releasing nanospheres to be aggregated (blue suspension). The measurable colorimetric change was used for quantification of CAP.

##### Performance of the Assay in Buffer

The ratios of plasmon absorbances of gold nanospheres at 610 nm and 520 nm (triplicate readings) for different concentrations of CAP were plotted and the linear range was determined. The limit of detection (LOD) and limit of quantification (LOQ) were ascertained using the following equations:

LOD = 3S_b_/m

LOQ = 10S_b_/m,

where S_b_ is the standard deviation of the signal for blank and m is the slope of the linear regression curve.

##### Validation in Real Samples

Five grams of honey were diluted with 15 mL water and divided into fifteen aliquots. Three aliquots for each of the five samples were analyzed—one without artificial contamination and four artificially contaminated samples with 10, 50, 100, and 1000 pg mL^−1^ CAP. The mixture was vortexed until it reached homogeneity and filtered (0.45 μm) to remove denatured proteins. The responses of the colorimetric biosensing were validated using HPLC analysis.

##### Reproducibility and Specificity

All experiments were conducted in triplicates on the same day. Intraday and interday precision were established by analyzing a single concentration of CAP in the buffer 10 times on a single day and once every 10 consecutive days within the same laboratory. A selection of analogues of CAP (florfenicol, thiamphenicol, CAP succinate at 100 ng/mL concentration in buffer) were subjected to the aptasensing method to determine their specificities.

## 3. Results and Discussion

### 3.1. Truncation of the Original Aptamer

**Figure 1:**
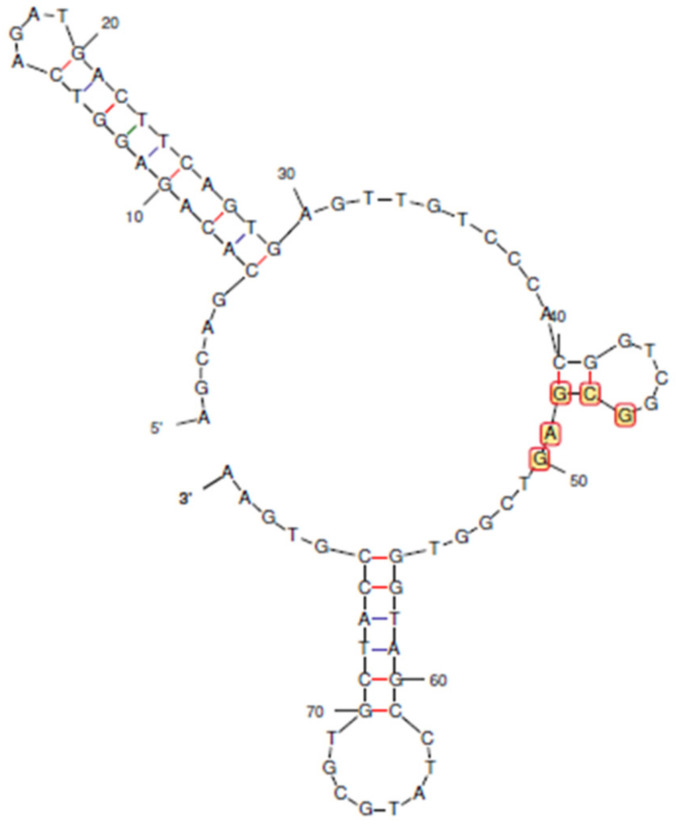
The predicted secondary structure of the reported aptamer for chloramphenicol. Bases 46 to 50 were found to be consensus regions for binding to CAP. Blue bonds indicate A-T base pairing, and red bonds indicate G-C base pairing.

Aptamers with sequence lengths between 40–50 bases have been successfully used for the biosensing of low molecular weight targets, such as tetracyclines and fungal toxins [32,33]. Overly long aptamers tend to bind non-specifically. CAP is a small molecule with a molar mass of 323 g/mol, whereas its reported aptamer is 80 bases long (Figure 1). Therefore, we attempted to truncate the aptamer, expecting to increase its binding affinity, as has been performed for other analytes.

The primer-binding terminal bases of original aptamer (Sequence 1) do not participate in secondary structures, whereas the consensus (46–50 bases) was essential [27]. Therefore, a rational truncation design was started with 5 bases (Sequences 2, 3, and 4), followed by 20 bases (Sequences 5, 6, and 7), 30 bases (Sequences 8, 9, and 10) from either or both terminals, and, finally, 40 bases only from the 5′ end (Sequence 11).

### 3.2. Determination of Secondary Structures of Truncated Oligonucleotides

For the aptamer to function as a specific receptor for binding, it must fold into a secondary conformation, which is a function of the nucleotide sequence. A reduction in length, therefore, changes the secondary structure and may affect molecular recognition. It was specifically necessary to predict if a hairpin bend in the binding consensus region would form [27].

**Figure 2:**
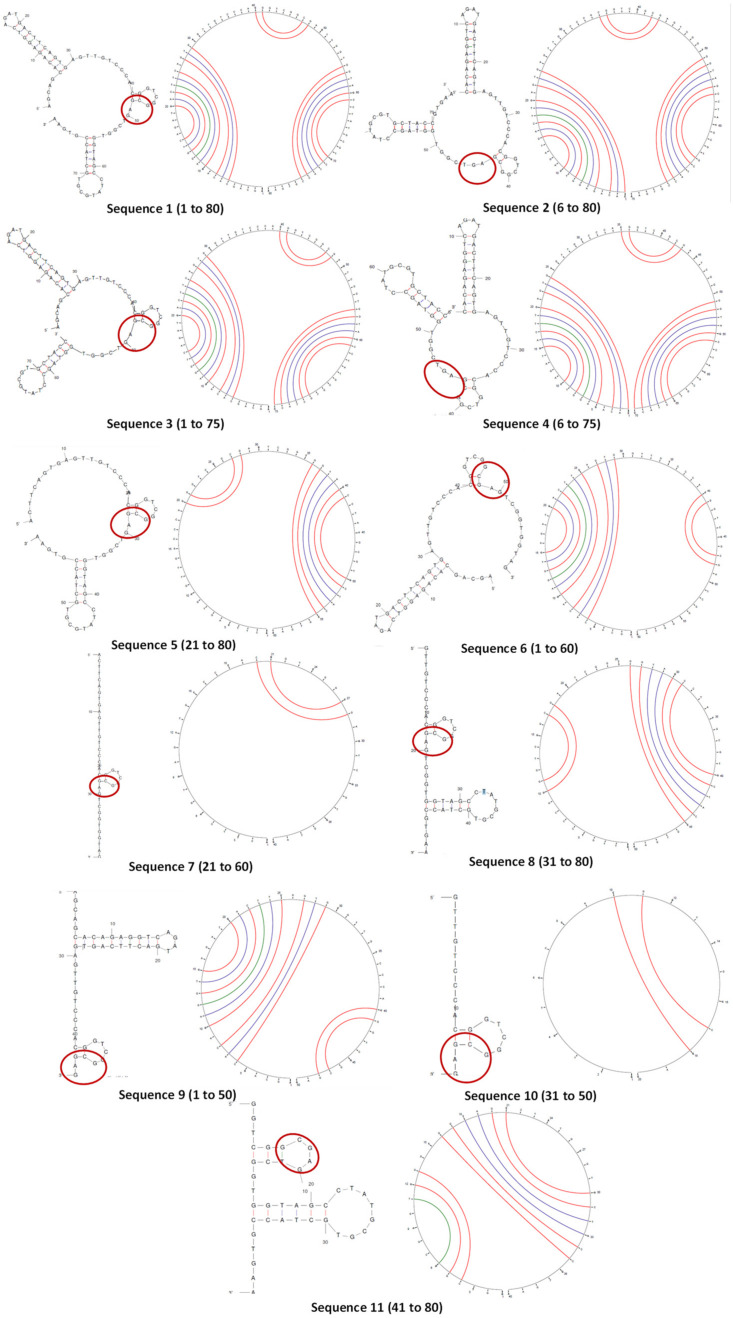
The folded secondary structures and circular plots of Sequences 1 to 11. The arcs in the circular plots join the bases that hybridize in the duplex structure. The consensus region in the secondary structures is circled in red.

The secondary structures and circular plots of the truncated oligonucleotides were generated using Mfold (Figure 2). Wherever multiple structures were generated, the most stable configuration (as inferred from the ΔG) was selected. It can be seen that the target-binding hairpin loop was present in all the structures, supported by the circular plots (two distinct cytosine–guanine triple bonds). This proves that the structure plays a crucial role in stability and recognition [34]. However, the other regions and their interactions varied largely with longer truncations (sequences 5 to 11). Sequences 7 and 10 did not form any other secondary structure except the hairpin.

### 3.3. Computational Prediction of Stability of Truncated Oligonucleotides

Stable folding relies on the energetics, that is, the ΔG and ΔS of the molecules, and the Tm of the duplexes [29,30]. These parameters are dependent on nucleotide base stacking, electrostatic repulsion between negative bases, rigidity of conformation, hydrogen bonding between complementary bases, and hydrogen bonding between nucleotides and solvent water [35].

**Figure 3:**
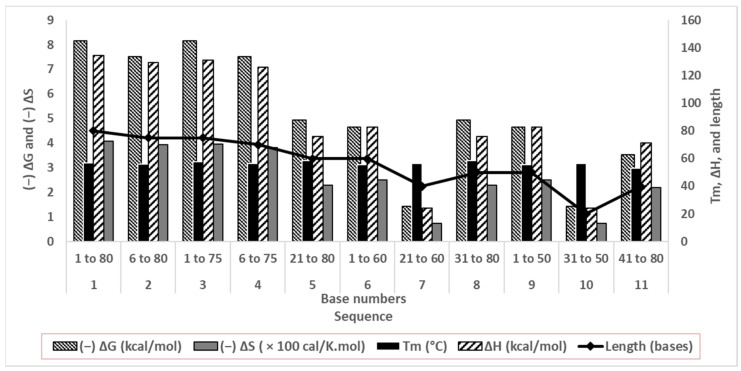
Thermodynamic parameters of folding of truncated oligonucleotides in correlation with their lengths.

Although structural stability does not directly affect aptamer affinity, this computational study corroborates our secondary structure predictions. We observed less change in the predicted thermodynamic properties where secondary structures were absent (Figure 3). A low ΔH de-stabilized base stacking and pairing, whereas less (−)ΔS change indicated insignificant conformational change upon the solvation of oligonucleotides in water.

Sequences 1 to 4 differed by 5 or 10 bases from each other, with similar predicted energetics and stable folding in the solution (a high negative ΔG and a less negative ΔS). Their patterns of and abilities to form duplexes were similar as well (computational Tm and structures are shown in Figure 2). Since the goal of truncating was to considerably shorten aptamers while retaining conformational stability, sequences 5 to 11 were minutely studied. The duplexes were quite stable, with Tm values between 53.1 and 58.5 °C. Sequences 5, 6, 8, and 9 showed quite high computed (−)ΔG and ΔH values, and comparatively lower (−)ΔS values, signifying good base stacking and solvation. For sequences 7, 10, and 11, ΔG and ΔH values decreased sharply, suggesting poor conformational stability. Sequences 7 and 11 had 40 bases, and Sequence 10 had only 20 bases, signifying that the drastic truncation of aptamers strongly affects their folding.

### 3.4. Binding Affinity with Analyte

The crucial factor of the aptamer–analyte binding affinity was studied next using BLI [36]. BLI offers an advantage over surface plasmon resonance or optical waveguide-based kinetic measurements, since it uses a static microfluidic system without requiring a sample flow across a sensor. A well-plate is agitated to mimic fluidics (Figure S1), leading to the simple, quick, and high-throughput measurement of kinetics [36,37].

**Figure 4:**
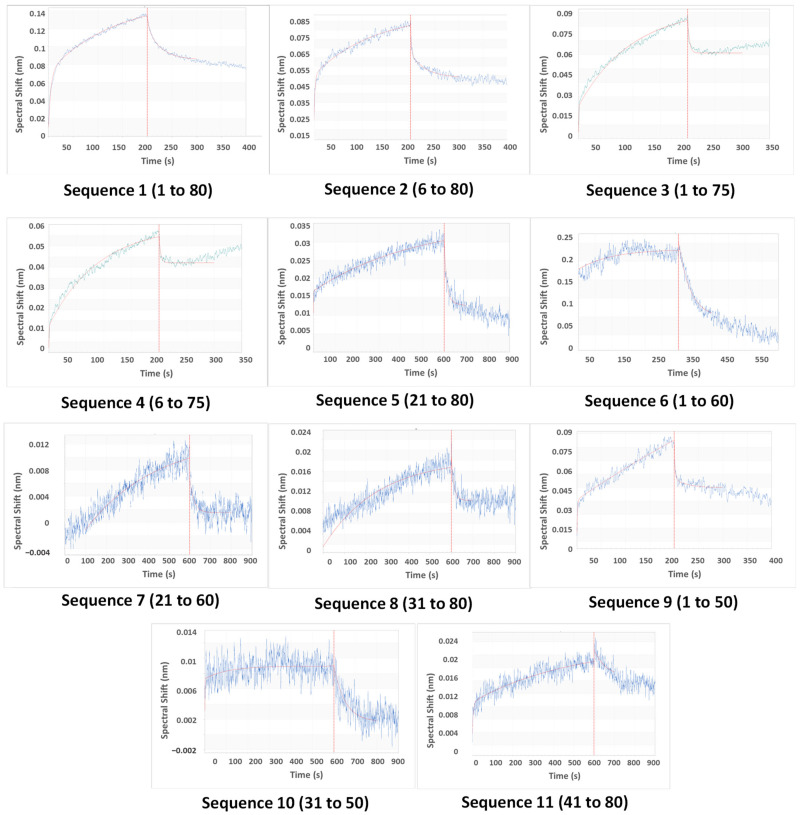
The fitted association-dissociation curves of the original and truncated oligonucleotides, as obtained using a biolayer interferometry assay (*y*-axis: spectral shift in nm, *x*-axis: time in s) through the Octet data acquisition software. The axis formats of the plots have been modified for better visibility. The unmodified software-acquired plots have been provided in the Supplementary Material.

The aptamer was first loaded onto probes (Figure S2), then CAP was allowed to attach to the aptamer (association) and then stripped off from it (dissociation) (refer to Supplementary Information). Since the binding capacities of oligonucleotides differ, the association time was varied to allow for complete capture (*x*-axis, Figure 4). For the same reason, the magnitude of the shift was also varied (*y*-axis, Figure 4). The best fit among replicates was selected. The data were subjected to local curve fitting (shown in red) following the 1:1 stoichiometric model. The choice of the final oligonucleotide was based on the regression coefficient of the fitting (R^2^) and K_d_ (Table 2).

The original aptamer curve (Sequence 1) and sequences of similar lengths (2, 3, and 4) showed a high magnitude of shift and low noise. However, both the association and dissociation curves in these four cases had low slopes. These slopes are rate constants that are numerically denoted as k_assoc_ (association) and k_dissoc_ (dissociation). Low slopes denote that both binding and dissociation are gradual. The K_d_ values (which are ratios of k_dissoc_ and k_assoc_) of Sequences 1 to 4 are higher as a result of low k_assoc_.

A considerable difference in curve patterns was observed for longer truncation. The removal of 20, 30, and 40 bases reduced the shift and introduced noise. Association and dissociation curves were steeper in some (Sequences 6 and 8), but few were deviant from the model (low R^2^). However, our goal was not only to lower K_d_ but also to reduce the aptamer length, so that aptamer does not bind strongly to both CAP and competing species. Therefore, the choice of aptamers was made from Sequences 4 to 11. Sequence 9 was the best choice, as the BLI curve showed lower noise, better R^2^, and steeper association and flatter dissociation, giving a K_d_ of 0.092 μM. However, Sequence 9 was still 50 bases long, and it was important to check if similar and shorter oligonucleotides (7, 8, 10, and 11) displayed appreciable aptasensing efficiencies. Sequence 10 bound weakly (low K_d_ and R^2^), so it was eliminated from the study at this point. Sequences 7, 8, 9, and 11 were investigated for analytical performance.

### 3.5. Aptasensing of CAP

#### 3.5.1. Performance of the Assay

**Figure 5:**
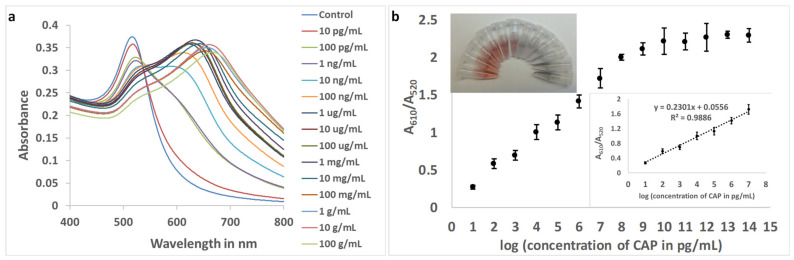
(**a**): Absorbance spectra of aptamer (Sequence 9)-gold nanoparticles with increasing concentrations of CAP in buffer. (**b**) The plot of the ratio of absorbance at 610 nm and 520 nm. (**Inset top**: Photograph of color change in gold nanocolloid with increasing concentrations of CAP. **Clockwise**: Control, 10 pg mL^−1^ to 100 mg mL^−1^, with intervals of one order of magnitude. **Inset bottom**: The linear range of response (n = 3)).

With the original aptamer (Sequence 1), the resolution of the assay was unsatisfactory, and 299.64 pg mL^−1^ of CAP in the buffer could be accurately quantified (Figure S3). With Sequence 9 and optimized concentrations, the response (A_610_/A_520_) was plotted against the logarithmic values of CAP concentrations in the buffer. For the improved aptamer (Sequence 9), the linear range was found to be from 10 pg mL^−1^ to 10 μg mL^−1^, with a resolution of 1 order of magnitude in concentration (Figure 5).

The LOD and LOQ for the improved aptasensing were estimated to be 1.673 pg mL^−1^ (parts per trillion) and 5.563 pg mL^−1^, respectively, in the buffer with a 9.8% standard deviation of blank values for triplicate readings. The improvement in LOD, achieved using aptamer truncation, was calculated to be 179.10 times.

As observed in Figure 5b (inset: photograph), the change in color of the nanosphere colloid with the addition of CAP was visually identifiable. The purpose of rapid visual tests is to enable farmers, regulatory authorities, and consumers to identify the presence of contaminants in food and the environment without the need for sophisticated instruments and dedicated personnel. This test will allow a field-applicable qualitative visual assay for CAP present at a minimum concentration of 10 ng mL^−1^. Additionally, limited quantification can be achieved, facilitating the primary screening of the antibiotic. Figure 5b (inset: photograph) shows that, within the linear range of the response curve, the bright red color of the colloid gradually changed into a purple and then bluish color. The naked eye will be able to distinguish concentrations that differ by at least three orders of magnitude, i.e., 10 pg mL^−1^, 10 ng mL^−1^, and 10 μg mL^−1^.

The performances of Sequences 7, 8, and 11 were determined via the quantifying recovery of different concentrations of CAP added to the buffer (Table S1). Sequence 8 could sense CAP at lower concentrations (=10 pg mL^−1^), while the others could not accurately detect CAP at ultrasensitive levels (>100 pg mL^−1^). This clearly showed that the length (50 bases) and structure were important for binding, and shorter sequences (40 and 20 bases) lacked high analytical potential. Such activity may be attributed to factors such as conserved bases, type and number of secondary structures, and length. The original report on Sequence 1 mentioned that, besides the consensus, there were two other discrete nucleotides that were conserved across the chosen aptamers [27]. These were guanine in the 27th position and adenine in the 39th position. Truncation resulted in the removal of guanine in Sequences 8 and 10 and both guanine and adenine in Sequence 11.

#### 3.5.2. Validation in Real Samples

The colorimetric method was proven to be capable of the ultrasensitive and accurate detection of CAP in real matrices. The accuracy was validated using the standard HPLC method (Table 3). Recovery values ranged from 94.7 to 100.4%, with standard deviations in the range 3.8 to 8.6%.

#### 3.5.3. Specificity and Reproducibility

The relative standard deviation of the responses from the reproducibility investigation was found to be 2.44% for intraday precision and 4.12% for interday precision, proving that the aptasensor was reproducible and stable.

**Figure 6:**
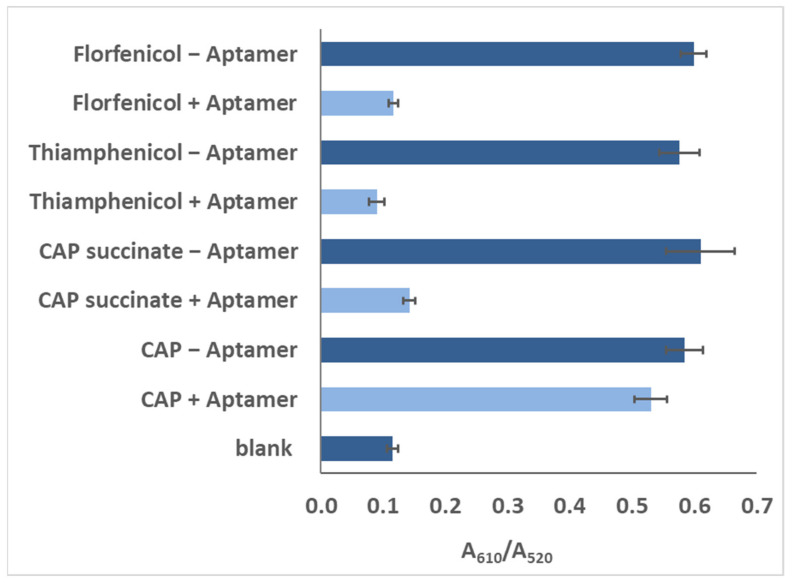
Responses of blank, 100 pg/mL of CAP, florfeniol, thiamphenicol, and CAP succinate, as recorded for the colorimetric assay with Sequence 9.

Different analogues of CAP in the buffer were subjected to the same bioanalysis, and results showed a high specificity for CAP (Figure 6), which may also be a result of stronger binding using the truncated aptamer (Sequence 9).

#### 3.5.4. Comparison with Reported Literature

A comprehensive compilation of reports published in the last five years on the colorimetric aptasensing of CAP is presented in Table S2. The aptamers used in most of these research works were adapted from the original reported sequence, with the primer binding bases removed (see Sequence 7, Table 1). Unlike our work, these studies were not aimed at structural modification and binding. There are a few exceptions, such as Chang et al. [17], who used the full, 80-base Sequence 1 and obtained a detection limit of 7.11 ng mL^−1^; and Xie et al. [20], who used the de novo-selected shorter aptamer by Duan et al. [26], achieving a sensitivity of 8.1 ng mL^−1^. Our aptasensing method is among the most sensitive of the compiled reports (299.64 pg mL^−1^ with Sequence 1 and 1.673 pg mL^−1^ with Sequence 9). While using Sequence 7, the lowest concentration we could accurately detect (91.83% recovery) was 200 pg mL^−1^. Some authors [9,16] have reported lower LODs (0.30 and 0.13 pg mL^−1^ respectively), probably achieved via magnetic separation, target recycling, and DNAzyme-hemin catalysis. Many groups have attained comparable and appreciable limits, such as 20 [9], 3 [10], 15 [11], 20 [15], and 3.10 [18] pg mL^−1^, where the signal was amplified using HRP-enzyme substrate reaction and the capture/detection conjugate was enabled via cDNAs, single-stranded binding proteins, or antibodies. One particularly notable method [16] employed ssDNA-based aptamer locking to counter the effects of length and achieved a very high sensitivity of 9.69 pg mL^−1^. The work by Tao et al. 2020 [38] needs a special mention, as they made a similar attempt to truncate the CAP aptamer to 40 bases. The shortened aptamer, however, was not desorbed from gold nanospheres in the presence of CAP and, therefore, was not suitable for biosensing. The binding affinity could not be accurately measured using isothermal titration calorimetry. This study provides very useful insight into the choice of method used for K_d_ determination.

Several of the previously reported colorimetric aptasensing methods employed complicated, multi-step operations, two or more expensive biomolecules (complementary strands, chromogenic enzymes and substrates, antibodies, exonucleases, biotin-streptavidin, DNAzyme, single-stranded-binding proteins, hemin, etc.), and multiple sets of nanoparticles for capture and signal amplification. Given the need for the rapid, on-site detection of food and agricultural contaminants, a few methods are particularly significant [13,14,16,17,20,21]. Our method is among them, being one-step, simple, and ultrasensitive with qualitative visual analysis and quantification using a spectrophotometer or hand-held colorimeter.

## 4. Conclusions

A strong binding affinity between aptamer and analyte is imperative for biomonitoring. A lack of sensitivity in the nanosphere aggregation-based colorimetric aptasensing of CAP prompted the present investigation on truncating an aptamer to improve its binding affinity—a first report for the CAP aptamer. In silico evaluation and bio-layer interferometry showed that a 50-base oligonucleotide had stable configuration and stronger binding. This aptamer improved the detection limit by 32.87 times. Earlier literature on CAP aptasensing has employed multiple recognition, amplification, and capture molecules, as well as several steps and a complicated assay format. Our work is the first on the non-cross-linked aggregation of nanospheres for CAP detection. It boasts of simple and visual yet ultrasensitive detection. Given the serious threats posed by antibiotics in food and the necessity of a field-applicable, convenient analysis, ours is a promising biosensing technique. It will enable the identification of minute traces of the antibiotic, possibly facilitating the prevention of its entry into the food chain and subsequent health hazards.

## Figures and Tables

**Table 1:** Aptamer sequences with curtailed lengths ^a^.

Seq. No	Sequence (5′ 🡪 3′)	No. of Bases Removed	Terminal/Seq Base Numbers
1	***AGCAGCACAGAGGTCAGATG***ACTTCAGTGAGTTGTCCCACGGTCG**GCGAG**TCGGTGGTAG***CCTATGCGTGCTACCGTGAA*** **(original)**	0	- 1 to 80
2	***CACAGAGGTCAGATG***ACTTCAGTGAGTTGTCCCACGGTCG**GCGAG**TCGGTGGTAG***CCTATGCGTGCTACCGTGAA***	5	5′ 6 to 80
3	***AGCAGCACAGAGGTCAGATG***ACTTCAGTGAGTTGTCCCACGGTCG**GCGAG**TCGGTGGTAG***CCTATGCGTGCTACC***	5	3′ 1 to 75
4	***CACAGAGGTCAGATG***ACTTCAGTGAGTTGTCCCACGGTCG**GCGAG**TCGGTGGTAG***CCTATGCGTGCTACC***	10	5′and 3′ 6 to 75
5	ACTTCAGTGAGTTGTCCCACGGTCG**GCGAG**TCGGTGGTAG***CCTATGCGTGCTACCGTGAA***	20	5′ 21 to 80
6	***AGCAGCACAGAGGTCAGATG***ACTTCAGTGAGTTGTCCCACGGTCG**GCGAG**TCGGTGGTAG	20	3′ 1 to 60
7	ACTTCAGTGAGTTGTCCCACGGTCG**GCGAG**TCGGTGGTAG	40	5′ and 3′ 21 to 60
8	GTTGTCCCACGGTCG**GCGAG**TCGGTGGTAG***CCTATGCGTGCTACCGTGAA***	30	5′ 31 to 80
9	***AGCAGCACAGAGGTCAGATG***ACTTCAGTGAGTTGTCCCACGGTCG**GCGAG**	30	3′ 1 to 50
10	GTTGTCCCACGGTCG**GCGAG**	60	5′ and 3′ 31 to 50
11	GGTCG**GCGAG**TCGGTGGTAG***CCTATGCGTGCTACCGTGAA***	40	5′ 41 to 80

^a^ Bold underlined bases signify the 5′-base consensus sequence that binds to CAP. Bold and italic bases at 5′ and 3′ ends are primer binding sites for SELEX protocol. The rest are random sequences for the SELEX library.

**Table 2:** Characteristics of the aptamer–analyte binding and curve fitting using bio-layer interferometry.

Sequence Number	K_d_ (μM)	R^2^
1	0.704 (previously reported value in literature is 0.766)	0.993
2	0.877	0.973
3	0.645	0.946
4	0.661	0.945
5	1.712	0.924
6	4.014	0.905
7	0.370	0.882
8	0.153	0.853
9	0.092	0.914
10	3.109	0.699
11	0.426	0.757

**Table 3:** Recoveries of CAP from spiked honey samples determined using the truncated aptamer (Sequence 9)-mediated colorimetric method (n = 3).

Spiked Amount (pg mL^−1^)	Aptasensing (pg mL^−1^)/(pg g^−1^)	HPLC (pg mL^−1^)/(pg g^−1^)	Recovery (%) ± SD (with Respect to Spiked Amount)
0	Not detected	Not detected	-
10	9.7/28.9	Not detected	96.6 ± 2.7
100	94.8/284.3	Not detected	94.7 ± 3.8
1000	1004.3/3012.8	Not detected	100.4 ± 6.9
10,000	9812.5/29,437.5	9766.7/29,300.0	98.12 ± 1.1
50,000	49,605.7/148,817.1	48,942.9/148,828.8	97.88 ± 8.6

## Data Availability

Data will be available on request.

## Supplementary Materials

The following supporting information can be downloaded at: https://www.mdpi.com/article/10.3390/bios13060660/s1. Figure S1. Design of experiment on the microplate for biolayer interferometry assay; Figure S2. The loading curves for ten sequences; Figure S3. Chloramphenicol detection using original long aptmaer : plot of the ratio of absorbance at 610 nm and 520 nm for different analyte concentration; Figure S4. Transmission Electron Micrograph images of synthesized gold nanospheres; Table S1. Comparison of detection recoveries by aptamer sequences 7, 8, 9 and 11; Table S2. Previously reported colorimetric aptasensing of CAP. References [39,40] are cited in Supplementary Materials.

## References

[1] Li J., Shao B., Shen J., Wang S., Wu Y. (2013). Occurrence of chloramphenicol-resistance genes as environmental pollutants from swine feedlots. Environ. Sci. Technol..

[2] (1994). Extralabel Drug Use in Animals. Fed. Regist..

[3] Durham M. (2004). A bitter taste of honey. The Guardian (International Edition).

[4] Moudgil P., Bedi J.S., Aulakh R.S., Gill J.P.S., Kumar A. (2019). Validation of HPLC Multi-residue Method for Determination of Fluoroquinolones, Tetracycline, Sulphonamides and Chloramphenicol Residues in Bovine Milk. Food Anal. Methods.

[5] FSSAI (2012). Manual of Methods of Analysis Food.

[6] Panel E., Chain F. (2014). Scientific Opinion on Chloramphenicol in food and feed. EFSA J..

[7] Storey J., Pfenning A., Turnipseed S., Nandrea G., Lee R., Burns C., Madson M. (2003). Determination of chloramphenicol residues in shrimp and crab tissues by electrospray triple quadrupole LC/MS/MS. Laboratory Information Bulletin 4306 Chloramphenicol Residues Shrimp Crab Tissues, Volume 2004, no. 26/03/2004. https://citeseerx.ist.psu.edu/document?repid=rep1&type=pdf&doi=a7afb82606d443adda9924fd503250125105e4fa.

[8] Sharma R., Raghavarao K.S.M.S. (2019). Nanoparticle-Based Aptasensors for Food Contaminant Detection. Nanomaterials for Food Applications.

[9] Gao H., Gan N., Pan D., Chen Y., Li T., Cao Y., Fu T. (2015). A sensitive colorimetric aptasensor for chloramphenicol detection in fish and pork based on the amplification of a nano-peroxidase-polymer. Anal. Methods.

[10] Gao H., Pan D., Gan N., Cao J., Sun Y., Wu Z., Zeng X. (2015). An aptamer-based colorimetric assay for chloramphenicol using a polymeric HRP-antibody conjugate for signal amplification. Microchim. Acta.

[11] Huang W., Zhang H., Lai G., Liu S., Li B., Yu A. (2019). Sensitive and rapid aptasensing of chloramphenicol by colorimetric signal transduction with a DNAzyme-functionalized gold nanoprobe. Food Chem..

[12] Li J., Yu C., Wu Y.N., Zhu Y., Xu J., Wang Y., Wang H., Guo M., Li F. (2019). Novel sensing platform based on gold nanoparticle-aptamer and Fe-metal-organic framework for multiple antibiotic detection and signal amplification. Environ. Int..

[13] Wu Y.y., Liu B.w., Huang P., Wu F.Y. (2019). A novel colorimetric aptasensor for detection of chloramphenicol based on lanthanum ion–assisted gold nanoparticle aggregation and smartphone imaging. Anal. Bioanal. Chem..

[14] Miao Y., Gan N., Li T., Zhang H., Cao Y., Jiang Q. (2015). A colorimetric aptasensor for chloramphenicol in fish based on double-stranded DNA antibody labeled enzyme-linked polymer nanotracers for signal amplification. Sens. Actuators B Chem..

[15] Miao Y., Gan N., Ren H.X., Li T., Cao Y., Hu F., Yan Z., Chen Y. (2015). A triple-amplification colorimetric assay for antibiotics based on magnetic aptamer-enzyme co-immobilized platinum nanoprobes and exonuclease-assisted target recycling. Analyst.

[16] Abnous K., Danesh N.M., Ramezani M., Emrani A.S., Taghdisi S.M. (2016). A novel colorimetric sandwich aptasensor based on an indirect competitive enzyme-free method for ultrasensitive detection of chloramphenicol. Biosens. Bioelectron..

[17] Chang C.C., Wang G., Takarada T., Maeda M. (2017). Iodine-mediated etching of triangular gold nanoplates for colorimetric sensing of copper ion and aptasensing of chloramphenicol. ACS Appl. Mater. Interfaces.

[18] Luan Q., Xi Y., Gan N., Cao Y., Li T., Chen Y. (2017). A facile colorimetric aptamer assay for small molecule detection in food based on a magnetic single-stranded DNA binding protein-linked composite probe. Sens. Actuators B Chem..

[19] Javidi M., Housaindokht M.R., Verdian A., Razavizadeh B.M. (2018). Detection of chloramphenicol using a novel apta-sensing platform based on aptamer terminal-lock in milk samples. Anal. Chim. Acta.

[20] Xie Y., Huang Y., Tang D., Cui H., Cao H. (2018). A competitive colorimetric chloramphenicol assay based on the non-cross-linking deaggregation of gold nanoparticles coated with a polyadenine-modified aptamer. Microchim. Acta.

[21] Yan C., Zhang J., Yao L., Xue F., Lu J., Li B., Chen W. (2018). Aptamer-mediated colorimetric method for rapid and sensitive detection of chloramphenicol in food. Food Chem..

[22] Sharma R., Ragavan K.V., Raghavarao K.S.M.S., Thakur M.S. (2016). Nano-aptamer Based Quantitative Detection of Chloramphenicol. Biotechnology and Biochemical Engineering.

[23] Thakur M.S., Ranjan R., Vinayaka A.C., Abhijith K.S., Sharma R. (2013). Nanoparticles and Biophotonics as Efficient Tools in Resonance Energy Transfer-Based Biosensing for Monitoring Food Toxins and Pesticides. Adv. Appl. Nanotechnol. Agric..

[24] Mittal R., Sharma R., Ozturk M., Roy A., Bhat R.A., Vardar-Sukan F., Tonelli F.M.P. (2023). Phyconanofabrication-algae as bio- templates for commercially applicable nanomaterials. Synthesis of Bionanomaterials for Biomedical Applications.

[25] Mckeague M., Calzada V., Cerchia L., Derosa M., Heemstra J.M., Janjic N., Johnson P.E., Kraus L., Limson J., Mayer G. (2022). The minimum aptamer publication standards (MAPS guidelines) for de novo aptamer selection. Aptamers.

[26] Duan Y., Gao Z., Wang L., Wang H., Zhang H., Li H. (2016). Selection and Identification of Chloramphenicol-Specific DNA Aptamers by Mag-SELEX. Appl. Biochem. Biotechnol..

[27] Mehta J., Van Dorst B., Rouah-Martin E., Herrebout W., Scippo M.L., Blust R., Robbens J. (2011). In vitro selection and characterization of DNA aptamers recognizing chloramphenicol. J. Biotechnol..

[28] Zuker M. (2003). Mfold web server for nucleic acid folding and hybridization prediction. Nucleic Acids Res..

[29] Searle M.S., Williams D.H. (1993). On the stability of nucleic acid structures in solution: Enthalpy-entropy compensations, internal rotations and reversibility. Nucleic Acids Res..

[30] Owczarzy R., You Y., Groth C.L., Tataurov A.V. (2011). Stability and mismatch discrimination of locked nucleic acid-DNA duplexes. Biochemistry.

[31] Gao Y., Torrente-Murciano L. (2020). Mechanistic insights of the reduction of gold salts in the Turkevich protocol. Nanoscale.

[32] Yang Y., Yin Y., Li X., Wang S., Dong Y. (2020). Development of a chimeric aptamer and an AuNPs aptasensor for highly sensitive and specific identification of Aflatoxin B1. Sens. Actuators B Chem..

[33] Alawad A., Istamboulié G., Calas-Blanchard C., Noguer T. (2019). A reagentless aptasensor based on intrinsic aptamer redox activity for the detection of tetracycline in water. Sens. Actuators B Chem..

[34] Sharma R., Akshath U.S., Bhatt P., Raghavarao K. (2019). Fluorescent aptaswitch for chloramphenicol detection–Quantification enabled by immobilization of aptamer. Sens. Actuators B Chem..

[35] Abnous K., Danesh N.M., Ramezani M., Taghdisi S.M., Emrani A.S. (2016). A novel electrochemical aptasensor based on H-shape structure of aptamer-complimentary strands conjugate for ultrasensitive detection of cocaine. Sens. Actuators B Chem..

[36] Auer S., Koho T., Uusi-Kerttula H., Vesikari T., Blazevic V., Hytönen V.P. (2015). Rapid and sensitive detection of norovirus antibodies in human serum with a biolayer interferometry biosensor. Sens. Actuators B Chem..

[37] Gandhi I., Narendran K., Jackson G.W. (2011). Rapid DNA Aptamer Binding Characterization and ELASA Development Using Biolayer Interferometry (BLI). https://www.basepairbio.com.

[38] Tao X., He F., Liu X., Zhang F., Wang X., Peng Y., Liu J. (2020). Detection of chloramphenicol with an aptamer-based colorimetric assay: Critical evaluation of specific and unspecific binding of analyte molecules. Mikrochim. Acta.

[39] Dayne D., Gandhi I., Jackson G.W., Kumaraswamy S., Silva C. (2012). Fortebio Interactions.

[40] Haiss W., Thanh N.T.K., Aveyard J., Fernig D.G. (2007). Determination of Size and Concentration of Gold Nanoparticles from UV−Vis Spectra. Anal. Chem.

