# Supplementary material for: Rational Truncation of Aptamer for Ultrasensitive Aptasensing of Chloramphenicol: Studies Using Bio-Layer Interferometry
Source: Biosensors (Basel). 2023 Jun 16;13(6):660. doi: 10.3390/bios13060660 (PMC10296281; doi:10.3390/bios13060660)
Supplement: Supplementary file 1 [file biosensors-13-00660-s001.zip › biosensors-2389482-supplementary.pdf]

Supplementary information

Rational truncation of aptamer for ultrasensitive aptasensing of chloramphenicol: studies using bio-layer interferometry

Richa Sharma [1,2], Monali Mukherjee [2,3], Praveena Bhatt [2,3] and K. S. M. S. Raghavarao [1,2,4,*]

[1] Department of Food Engineering, CSIR-Central Food Technological Research Institute (CFTRI), Mysore 570020, India; cftri.ftbe.richa@gmail.com
[2] Academy of Scientific and Innovative Research (AcSIR), Ghaziabad 201002, India; monali0812@gmail.com (M.M.); praveena@cftri.res.in (P.B.)
[3] Department of Microbiology and Fermentation Technology, CSIR-Central Food Technological Research Institute (CFTRI), Mysore 570020, India
[4] Department of Chemical Engineering, Indian Institute of Technology (IIT)-Tirupati, Tirupati 517619, India
* Correspondence: raghava@iittp.ac.in or ksmsraghavarao60@gmail.com; Tel.: +91-821-2513910; Fax: +91-821-2517233

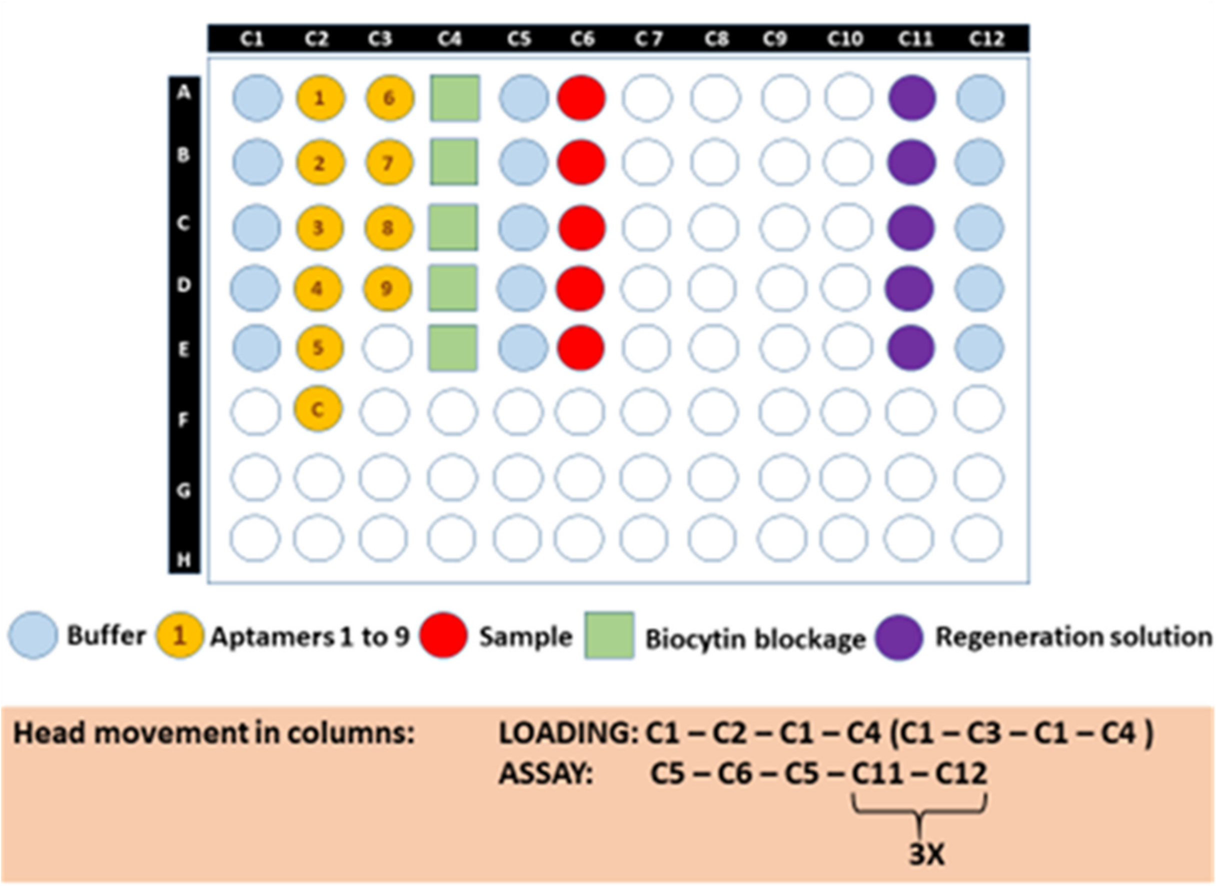

Figure S1. Design of experiment on the microplate for biolayer interferometry assay.

Detailed description of biolayer interferometry method for binding affinity

The first step is the loading of biotinylated truncated aptamers to the super-streptavidin (SSA) biosensor tips (100 nM of aptamers in 100 μL binding buffer). The loading step was optimized - SSA probes were allowed to dip in the wells containing aptamers for 600 seconds and subsequently in biocytin solution for 100 seconds to block vacant sites (Figure S2). CAP (1 μM in 100 μL binding buffer) was added in wells corresponding to the sensor probes. The sensors were dipped in the CAP solutions. Depending upon the association curve (binding rate), the association time was fixed. Since aptamers were different, this time varied from 150 to 600 seconds. The sensors were then placed in 100 μL binding buffer for 300 seconds for dissociation (the same well used for baseline is used for dissociation). In order to remove any remnant of CAP or other molecules, the probe was dipped in 100 μL regeneration solution for 5 seconds and then in 100 μL binding buffer for neutralization for another 5 seconds. These two sub-steps were alternately performed three times, making the total regeneration time 30 seconds. All the steps were accompanied by agitation for 30 secs at 1000 rpm speed for homogeneity. Baseline was run for 60 seconds.

The entire protocol was programmed into the Data Acquisition 9.0 software provided by the manufacturer. A double reference program was followed. The data from reference well (containing only buffer) was subtracted from the analyte wells for removing any buffer artefacts. The data from bare (without aptamer) probes were subtracted from the above result. The y-axis alignment, inter-step correction and Savitzky-Golay filtering were applied to the generated curves, for each sensor and sample well [39]. Finally, the processed data was allowed to fit the curve for association (entire association time was selected) and dissociation (first 100 seconds were selected) using 1:1 model with local fitting. The assay was performed in triplicates with same set of loaded aptamers.

The technology of BLI concerns the phenomenon of optical interferometry – the interaction of light waves. In the event of any molecule binding to the biolayer on a probe, a change in the interferometric profile will be observed. In the present case if the aptamer functionalised probe is dipped in CAP solution, CAP molecules bind and sinusoidal interferometric profile shifts. The magnitude of this shift (nm) increases with time resulting in an association curve. Similarly, if the probe now is dipped in a stripping solution, that is, one with a strong regenerating buffer, CAP desorbs gradually. The spectral shift magnitude reduces and gradually tends towards zero, as biolayer-aptamer approaches unbound state, giving a dissociation curve [39].

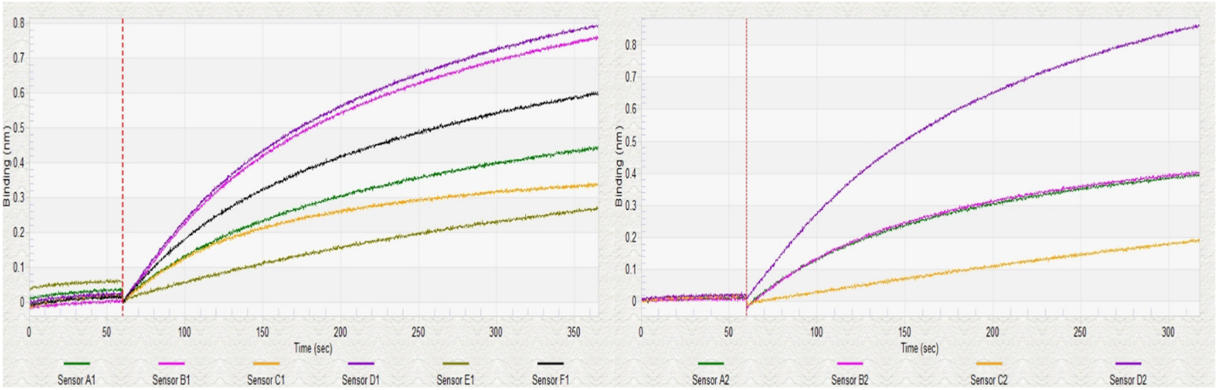

Figure S2. The loading curves for ten sequences (the original sequence loaded twice as control). The sensor names signify the probes to which each nucleotide is attached (Sensor A1,F1- 1 to 80, B1- 6 to 80, C1- 1 to 75, D1- 6 to 75, E1- 21 to 80, A2- 1 to 60, B2- 21 to 60, C2- 31 to 80, D2- 1 to 50).

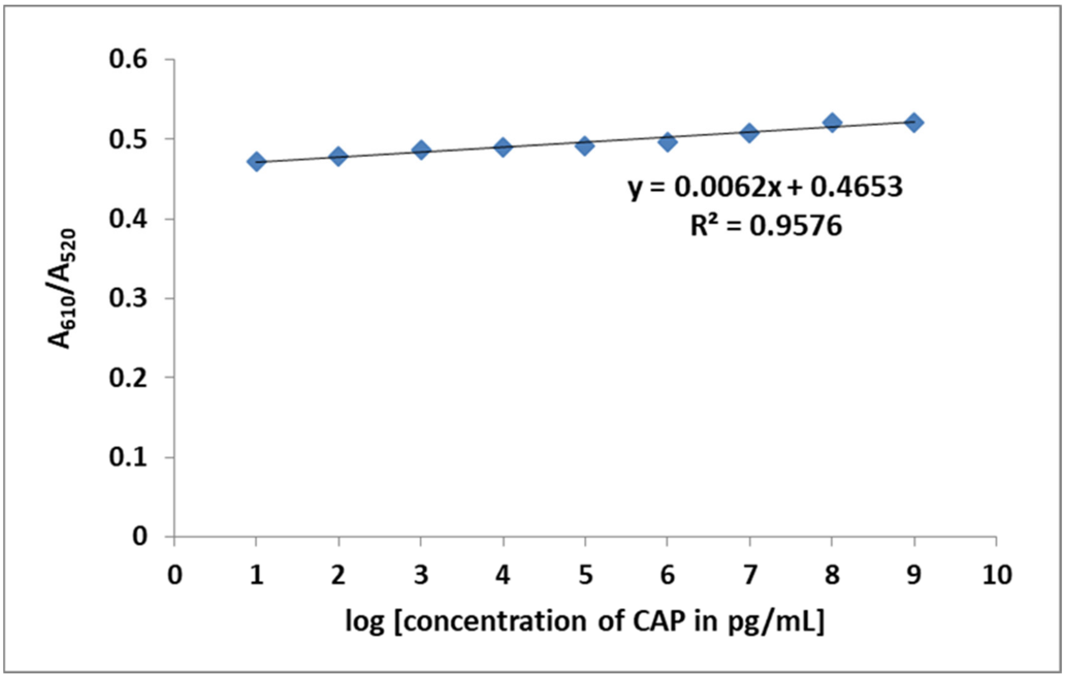

Figure S3. Chloramphenicol detection using original long aptmaer : plot of the ratio of absorbance at 610 nm and 520 nm for different analyte concentration. The limit of detection was calculated to be 299.64 pg mL^{-1} (standard deviation of blank was 1.1%).

Synthesis of gold nanospheres

Gold nanospheres were synthesized in lab.

Nanospheres were spectrally analysed by spectrophotometer UV-1601 (Shimadzu Scientific Instruments, USA). Further characterization was carried out using Transmission Electron Microscopy (Titan Themis 300kV, ThermoFisher Scientific, USA). The absorption maxima value for the synthesized colloid was measured to be at 516 nm, the average size in transmission electron microscopy was 27 nm and concentration was determined to be 1.7 x 10^{-9} M [40]

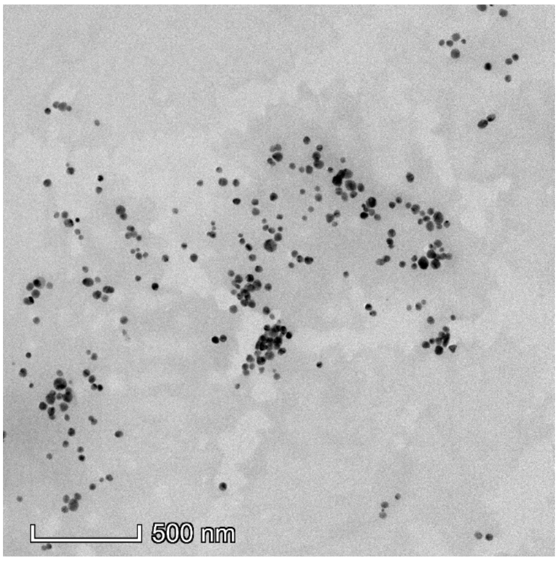

Figure S4. Transmission Electron Micrograph images of synthesized gold nanospheres

Table S1. Comparison of detection recoveries by aptamer sequences 7, 8, 9 and 11.

CAP added (pg mL^{-1})	Sequence 7		Sequence 8		Sequence 9		Sequence 11	
	Detected[a]	***Recovery***[b]	*Detected*	***Recovery***	*Detected*	***Recovery***	*Detected*	***Recovery***
1	-0.020	**-2.00**	0.005	**0.50**	0.012	**1.20**	-0.070	**-7.00**
5	0.003	**0.06**	0.190	**3.80**	4.117	**82.34**	-0.033	**-0.66**
10	-0.060	**-0.60**	8.001	**80.01**	9.158	**91.58**	0.940	**9.40**
50	40.700	**81.40**	46.100	**92.20**	50.990	**101.98**	39.000	**78.00**
100	84.830	**84.83**	95.877	**95.88**	96.090	**96.09**	82.114	**82.11**
200	183.655	**91.83**	187.505	**93.75**	190.115	**95.06**	157.420	**78.71**
500	425.996	**85.20**	448.773	**89.75**	492.004	**98.40**	430.855	**86.17**

[a] Ratio of A_{610} to A_{520} was calculated from absorbance curve. The experimental ratio was subtracted from the blank ratio (solution without analyte) to get the value in 'Detected' column.

[b] ('Detected'/ 'CAP added')*100, units %

Table S2. Previously reported colorimetric aptasensing of CAP.

Sl. no.	Detection principle	Significant features	Limit of detection (pg mL^{-1})<br>Real sample	Reference (refer to main manu-script)
1	Competitive<br>Signalling molecule: TMB chromogen (enzymatic)	Capture probe attached to core-shell Fe@Au nanoparticles<br>CAP competes with HRP labelled probe<br>Requirement of functionalized core shell iron nanoparticles, gold nanoparticles, cDNA, HRP, substrates<br>Multistep detection with magnetic separation	20 (buffer)<br>Fish<br>Pork	9
2	Competitive<br>Signalling molecule: TMB chromogen<br>(enzymatic)	cDNA (complementary to aptamer) attached to Fe@Au nanoparticles.<br>In presence of CAP, HRP labelled probe (with antibody) attaches to cDNA.<br>Requirement of functionalized core-shell iron nanoparticles, cDNA, HRP, antibodies, substrates<br>Multistep detection with magnetic separation	3 (buffer)<br>Fish<br>Duck	10
3	Competitive<br>Signalling molecule: TMB chromogen<br>(enzymatic)	Aptamer attached to Fe@Au nanoparticles<br>In presence of CAP, HRP labelled probe (with antibody) detaches from aptamer<br>Requirement of functionalized core-shell iron nanoparticles, cDNA, HRP, antibodies, substrates<br>Multistep detection with magnetic separation	15<br>Fish	11
4	Competitive<br>Signalling molecule: TMB chromogen<br>(enzymatic)	Aptamer-cDNA-Pt-HRP conjugate attached to antibodies on Fe@Au nanoparticles<br>In presence of CAP, cDNA-Pt-HRP HRP probe detaches from aptamer<br>Exonuclease I used to cleave single strands releasing CAP (for recycling) and Pt-HRP (for signalling)<br>Requirement of functionalized core-shell iron nanoparticles, Pt nanoparticles, cDNA, HRP and Exo I, antibodies, substrates<br>Multistep detection with magnetic separation	0.30<br>Milk	12

		Recycling of target increases signal		
5	Competitive<br>Signalling molecule: Gold nanoparticles	Free biotinylated aptamer binds to BSA on solid support, streptavidin-modified-DNA-nanoparticle conjugate attaches giving red colour.<br>Colour fades when aptamer engaged by CAP<br>Requirement of solid support, modification of aptamer (thiol, biotin), DNA to bind nanoparticles	145.67 (buffer)<br>72.352 (milk)<br>194.123<br>(rat serum)	13
6	Protection of nanoparticles by aptamers<br>Signalling molecule: Gold nanoparticles	Aptamers protected triangular nanoparticles are not etched to spherical particles Cu2+-assisted I−mediated method<br>Simple one-step method with minimum components	1.62×10^6 (buffer)	14
7	Competitive<br>Signalling molecule: TMB chromogen<br>(enzymatic)	Single stranded binding protein attached to Fe@Au nanoparticles<br>Aptamer attached to SiO2@Au-HRP probe<br>In presence of CAP, probe detached from magnetic nanoparticle complex<br>Requirement of functionalized core-shell iron nanoparticles, functionalized core shell silica nanoparticles, SSB protein, HRP, substrates<br>Multistep detection with magnetic separation	20<br>Milk	15
8	Protection of nanoparticles by ssDNA<br>Signalling molecule: Gold nanoparticles	Aptamer locked by short ssDNA in absence of CAP, leaving gold nanoparticles free for salt-induced aggregation<br>Simple, one-step method, providing solution for long length of CAP aptamer	9.69 (buffer)<br>Milk	16
9	Competitive between CAP-base and CAP<br>Signalling molecule: Gold nanoparticles	Negatively charged aptamer-functionalized gold nanoparticles aggregate in presence of positive CAP-base<br>Presence of CAP leads to de-aggregation<br>Simple, one-step detection	7.11×10^3 (buffer)<br>Spiked environmental water	17
10	Competitive	cDNA capture probe bound to microplate	3.10	18

	Signalling molecule: TMB chromogen<br>(enzymatic)	Aptamer tagged with HRP<br>Requirement of binding to solid support by streptavidin, HRP, substrate	Honey<br>Fish	
11	Competitive<br>Signalling molecule: TMB chromogen<br>(enzymatic)	Magnetic bead functionalized with aptamer<br>cDNA-gold nanoparticle-hemin/G-quadruplex DNAzyme catalysed TMB conversion<br>Requirement of complex functionalization of gold nanoparticles, cDNA, hemin, DNAzyme, substrates<br>Multistep detection with magnetic separation	0.13 (buffer)<br>Milk	19
12	Competitive<br>Signalling molecule: TMB chromogen<br>(enzymatic)	Fe-based metal organic framework catalyses TMB conversion.<br>Catalysis reduced if gold nanoparticle-aptamer-CAP complex binds to it<br>Simple, easy transduction	8.1×10^3 (buffer)<br>Spiked tap water	20
13	Protection of nanoparticles by ssDNA<br>Signalling molecule: Gold nanoparticles	Lanthanide attaches to aptamer functionalized gold nanoparticles and assists aggregation<br>In presence of CAP, aptamer detaches from nanoparticles<br>Simple, one-step detection<br>Can be detected through instrument and smartphone imaging app	2.471×10^3 (spectrophoto-meter)<br>1.899×10^3 (smartphone app)<br>Solid milk<br>Chicken	21
14	Protection of nanoparticles by aptamers<br>Signalling molecule: Gold nanoparticles	Truncated aptamers bind to gold nanospheres in absence of CAP<br>Unbound nanospheres aggregate in presence of salt, changing colour of colloid	1.67 (buffer)<br>Honey	***Present work***

Fitted association and dissociation curves (individual images) for Figure 4 main manuscript

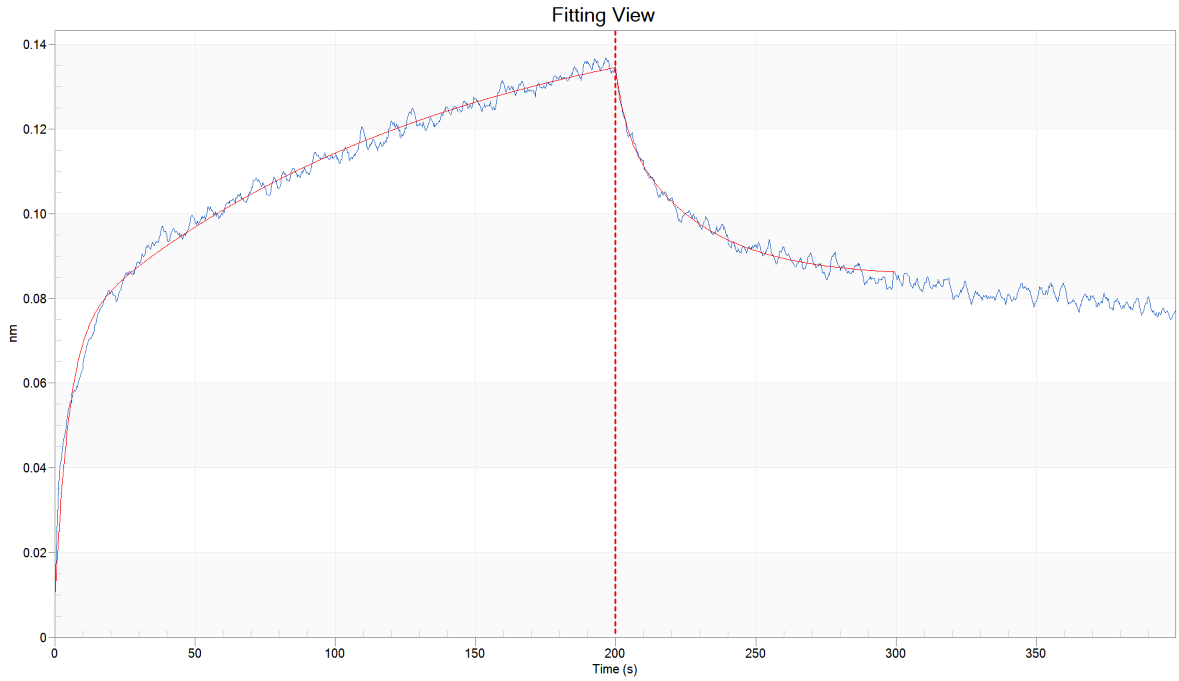

Sequence 1

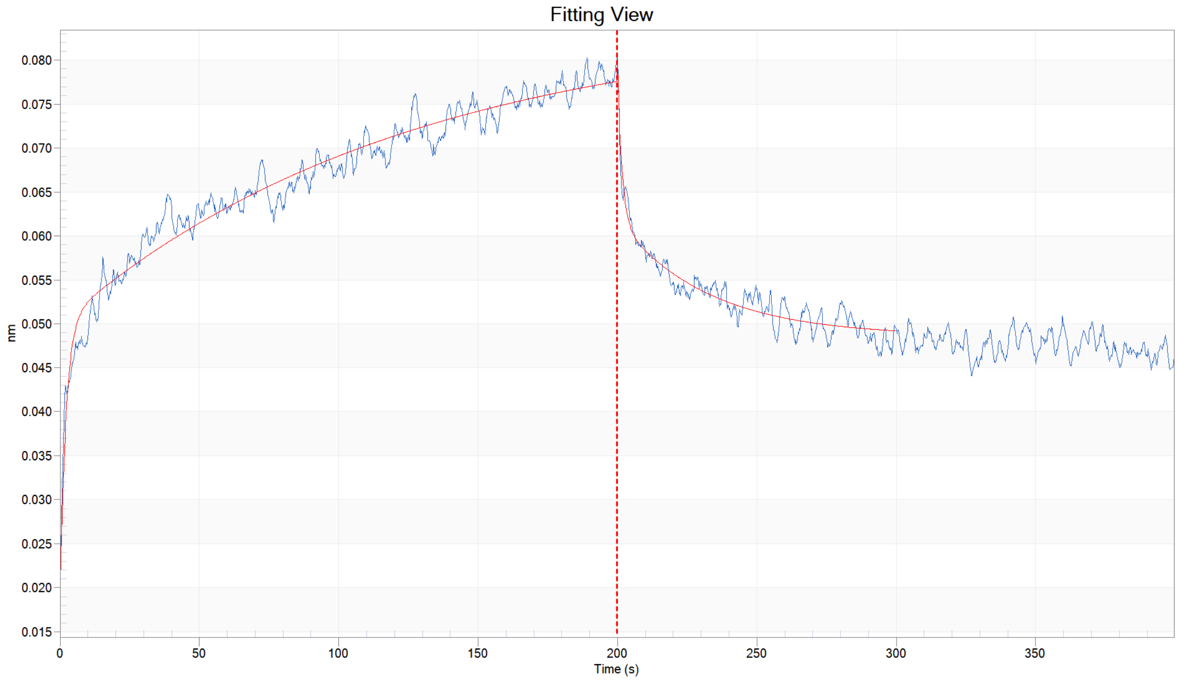

Sequence 2

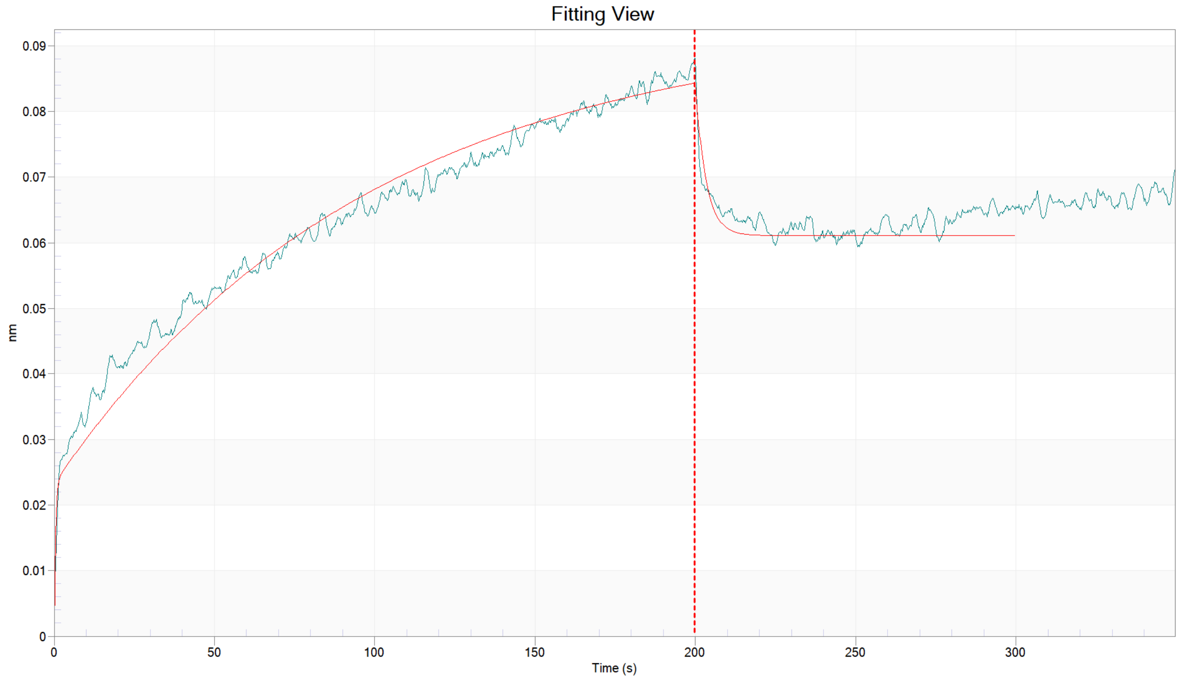

Sequence 3

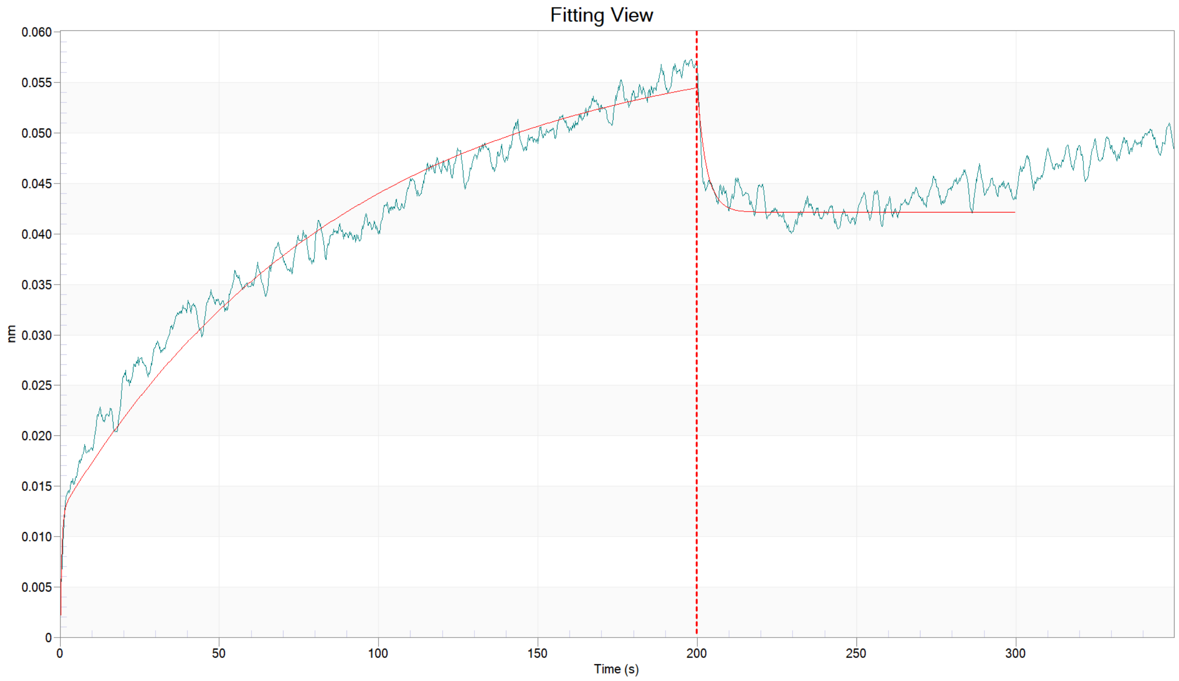

Sequence 4

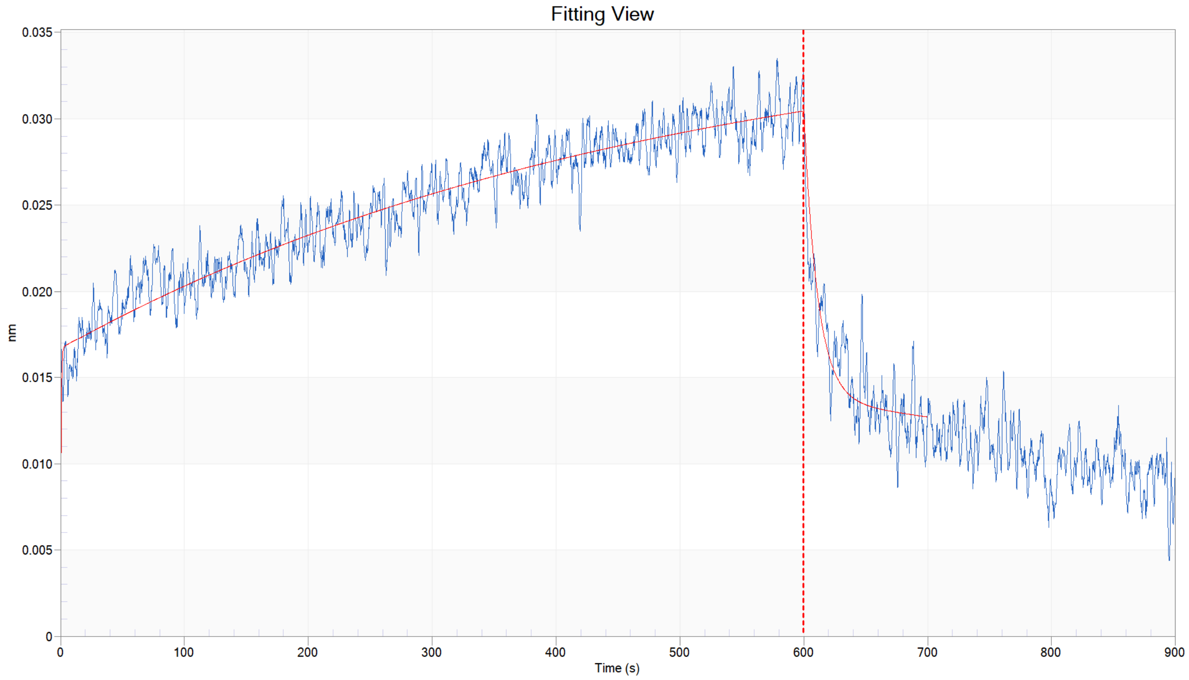

Sequence 5

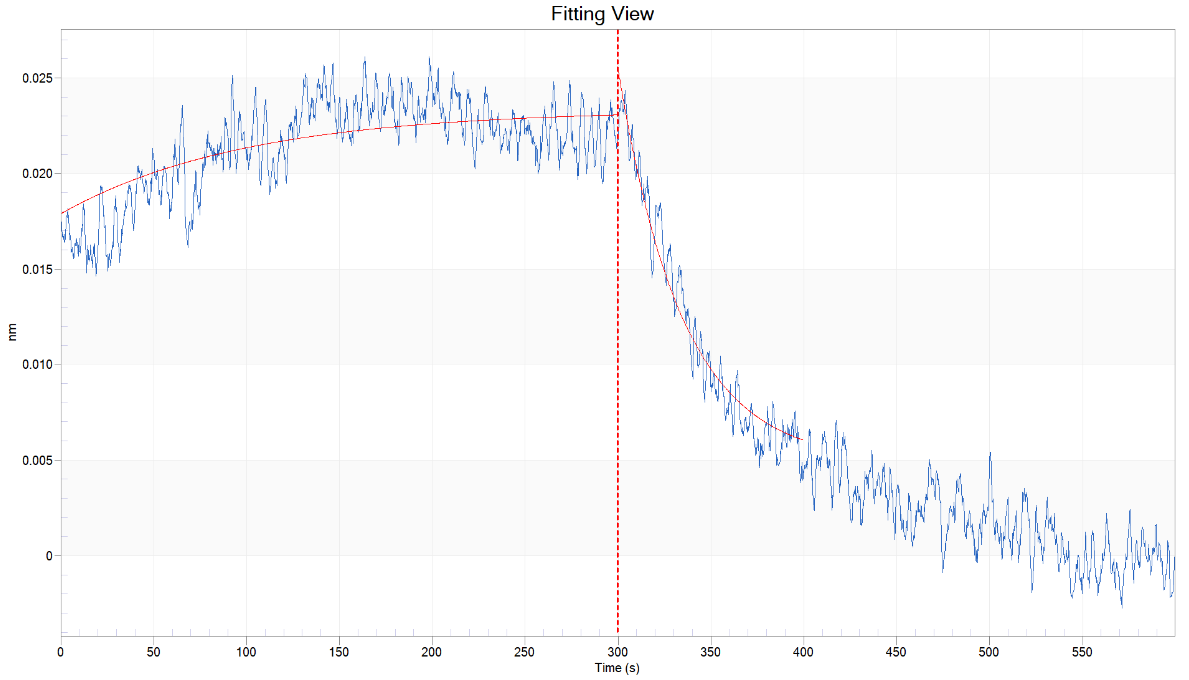

Sequence 6

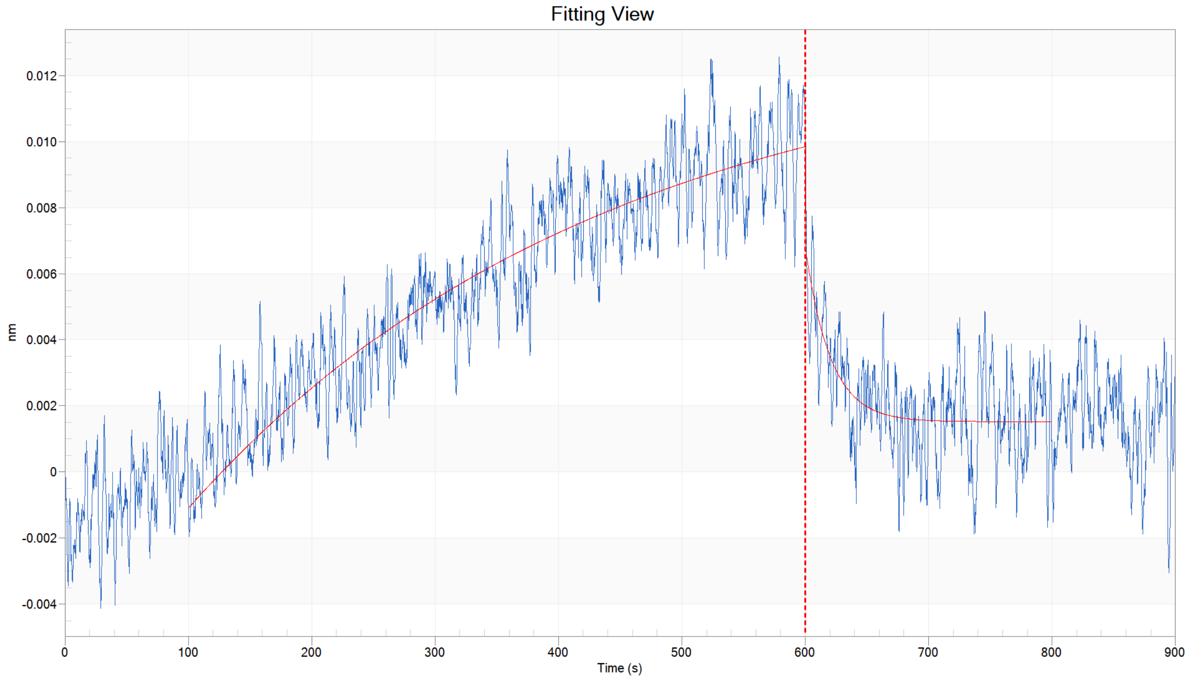

Sequence 7

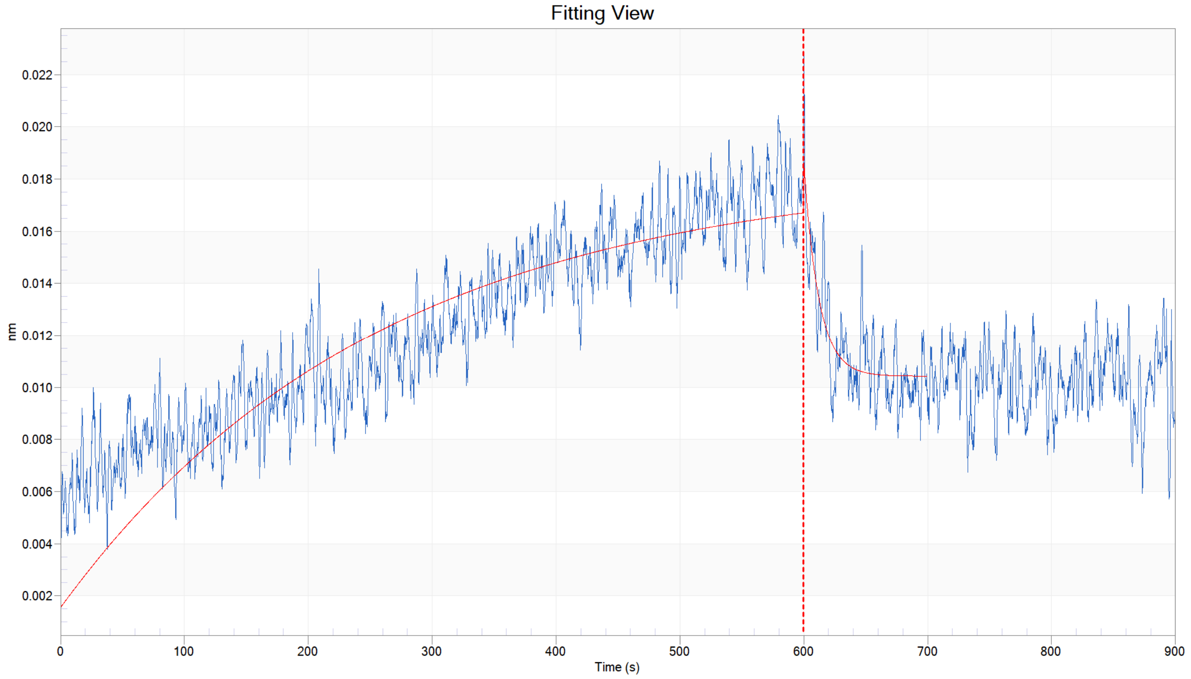

Sequence 8

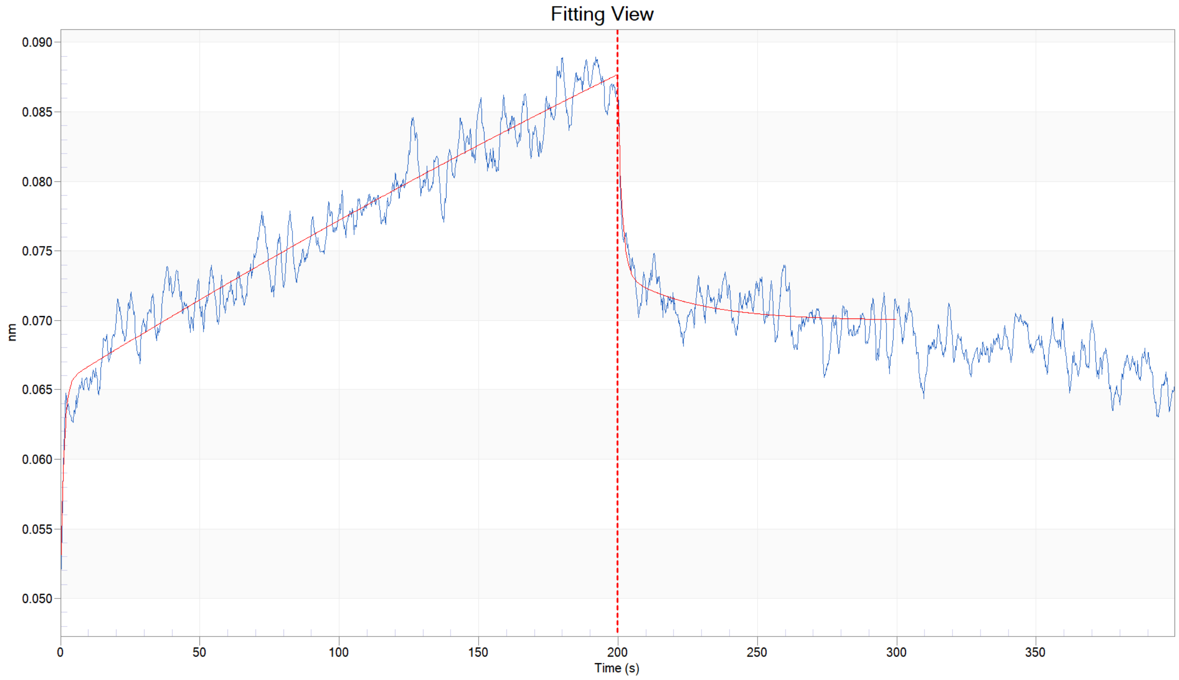

Sequence 9

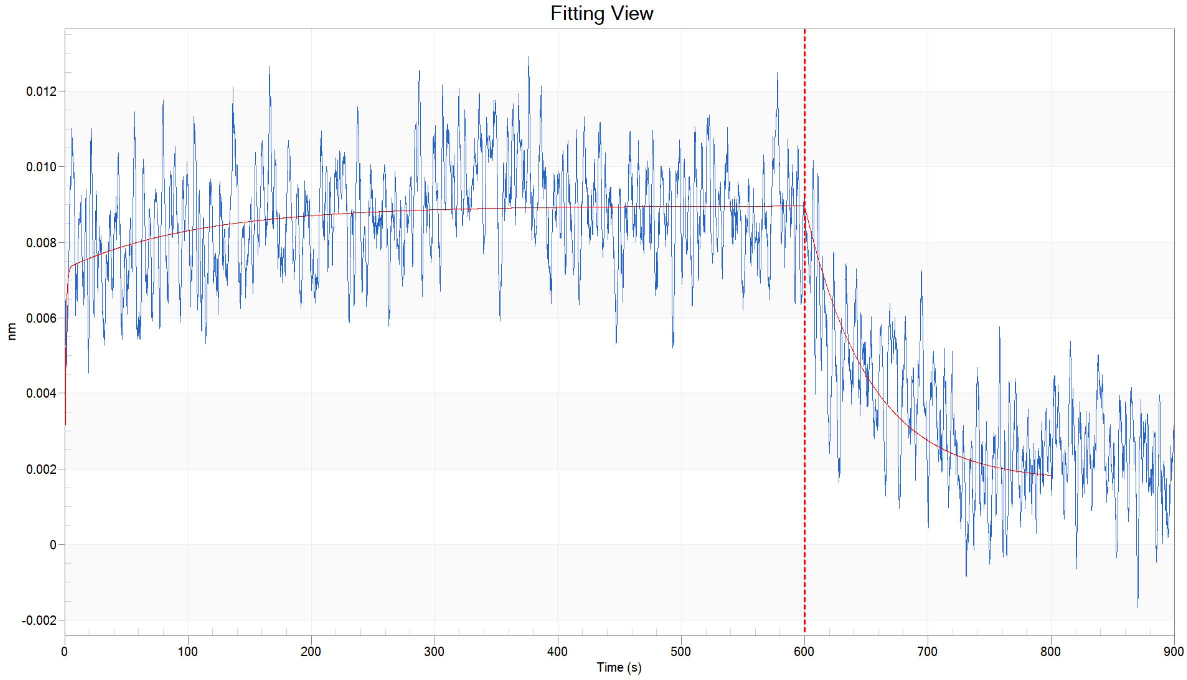

Sequence 10

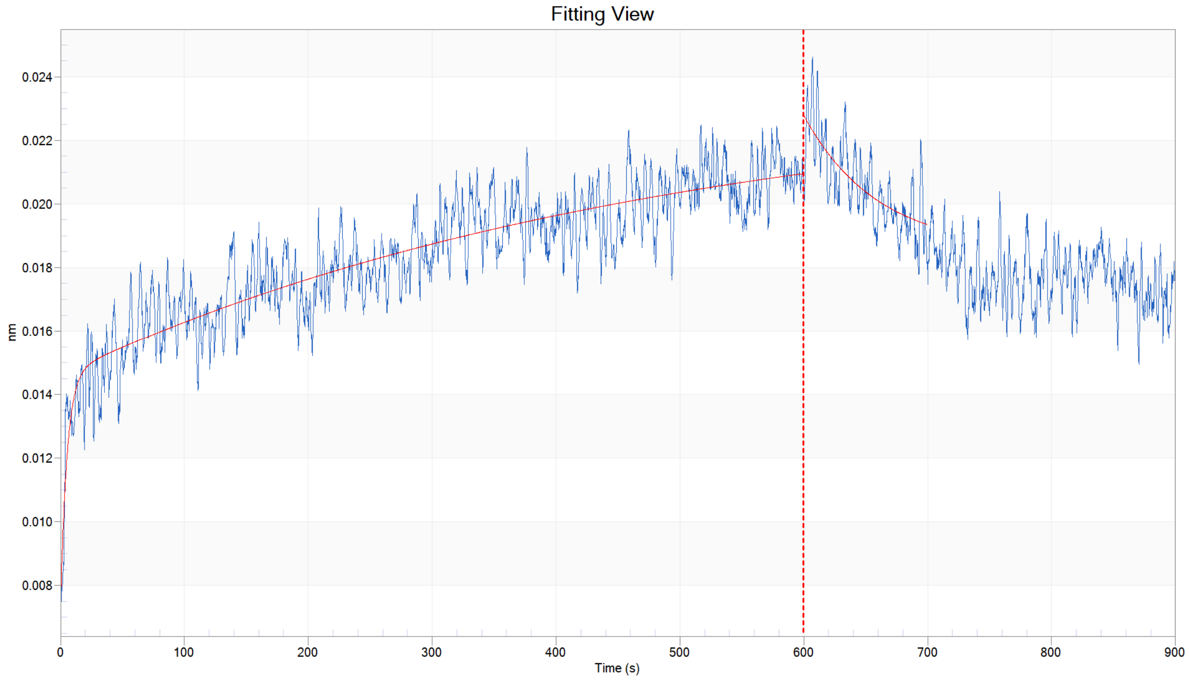
Fitting View
0.024
0.022
0.020
0.018
0.016
0.014
0.012
0.010
0.008
nm
0
100
200
300
400
500
600
700
800
900
Time (s)

Sequence 11
